# Podotimod in pediatric recurrent respiratory tract infections: a cost-utility analysis

**DOI:** 10.1186/s12890-022-02029-4

**Published:** 2022-06-23

**Authors:** Jefferson Antonio Buendía, Diana Guerrero Patiño, Erika Fernanda Lindarte

**Affiliations:** 1 Research Group in Pharmacology and Toxicology ”INFARTO”, Department of Pharmacology and Toxicology, University of Antioquia, Medellin, Colombia; 2 Research Group in Pharmacology and Toxicology ”INFARTO”, Department of Pharmacology and Toxicology, Facultad de Medicina, Universidad de Antioquia, Medellin, Colombia

**Keywords:** Health economics, Public health, Healthcare, Colombia, Corticosteroids

## Abstract

**Introduction:**

Despite the growing evidence on efficacy, few economic evaluations have evaluated the cost-utility of Pidotimod (PDT) supplementation to decrease the probability of recurrent respiratory tract infections in children. This study aimed to determine the cost-utility of PDT to reduce the incidence rate of recurrent respiratory tract infections in children.

**Methods:**

A decision tree model was used to estimate the cost and quality-adjusted life-years (QALYs) of PDT in a patient aged 1–6 with a history of recurrent respiratory tract infections. Multiple sensitivity analyses were conducted to evaluate the robustness of the model. Cost-effectiveness was evaluated at a willingness-to-pay (WTP) value of US$5180.

**Results:**

The base-case analysis showed that compared with placebo, PDT was associated with lower costs and higher QALYs. The expected annual cost per patient with PDT was US$797 (CI 95% US$794- US$801) and with placebo was US$1175 (CI 95% US$1169- US$1181). The QALYs per person estimated with PDT was 0.95 (CI 95% 0.94–0.95) and with placebo was 0.94 (CI 95% 0.94–0.94). The NMB with PDT was US$ 4121 (CI 95% 4114–4127) and with placebo was US$ 3710 (CI 95% 3700–3720). This position of absolute dominance (PDT has lower costs and higher QALYs than placebo) of PDT it is unnecessary to estimate the incremental cost-effectiveness ratio.

**Conclusion:**

In conclusion our study shows that PDT is a cost-effective strategy to reduce the incidence rate of recurrent respiratory tract infections in children. Our study provides evidence that should be used by decision-makers to improve clinical practice guidelines.

## Introduction

Recurrent respiratory tract infections (RTI) are frequent events that generate a high burden of morbidity in childhood. These RTI can affect about 25% of infants under 1 year old and 6% in the first 6 years of life [1]. Despite a benign condition that is likely to gradually improve by the age, RTI can cause significant medical, social, and economic problems for the child and society [1, 2]. RTI were associated with lower health-related quality of life in both children and their caregivers [3]. Children with RRTI showed significantly lower physical, emotional, social, and school functioning scores than healthy children and their caregivers lower scores on physical, emotional, social, cognitive, and communication functioning [3]. In addition to the preventive measures already proven to reduce the incidence of RRI, as reducing exposure to second-hand smoke, reducing exposure to indoor and outdoor pollutants, improve hand washing, promoting breastfeeding, adequate vaccination of children, bacterial immunomodulators have been proposed as preventive intervention [4].

Pidotimod (PDT, 3-L-pyroglutamyl-L-thiaziolidine-4-carboxylic acid) is a synthetic dipeptide molecule exerting effects on both innate and adaptive immunity [4]. In a recent systematic review and meta-analysis of 29 studies in 4344 children with RRI, PDT was associated with a significant increase in the proportion of participants who had lower RTIs (RR 1.59; 95% CI 1.45–1.74, *p* < 0.00001) compared with the conventional treatment. PDT could significantly decrease the duration of cough and fever and the number of patients in using antibiotics. Increased the levels of serum immunoglobulin (IgG, IgA, or IgM) and T-lymphocyte subtypes (CD3 + , CD4 +); without increasing the risk of other adverse events (RR = 1.05, 95% CI 0.72–1.54, *p* = 0.80) [5].

The Inter-society Consensus of Prevention of recurrent respiratory infections in 2021, regarding this drug states: “Pidotimod has demonstrated a consistent likelihood of efficacy and can be recommended in selected populations of children, always considering the cost–benefit ratio “[4]. However, to date, no economic evaluations have been published in developing countries. The contribution of an economic evaluation to the current evidence lies not only in estimating whether it is cost-effective, for example this kind of studies can estimate the cost-savings per patient treated with a new drug. The objective of the present study was to estimate the cost-utility of Pidotimod to reduce the incidence rate of recurrent respiratory tract infections in children.

## Materials and methods

### Base case

**Fig. 1:**
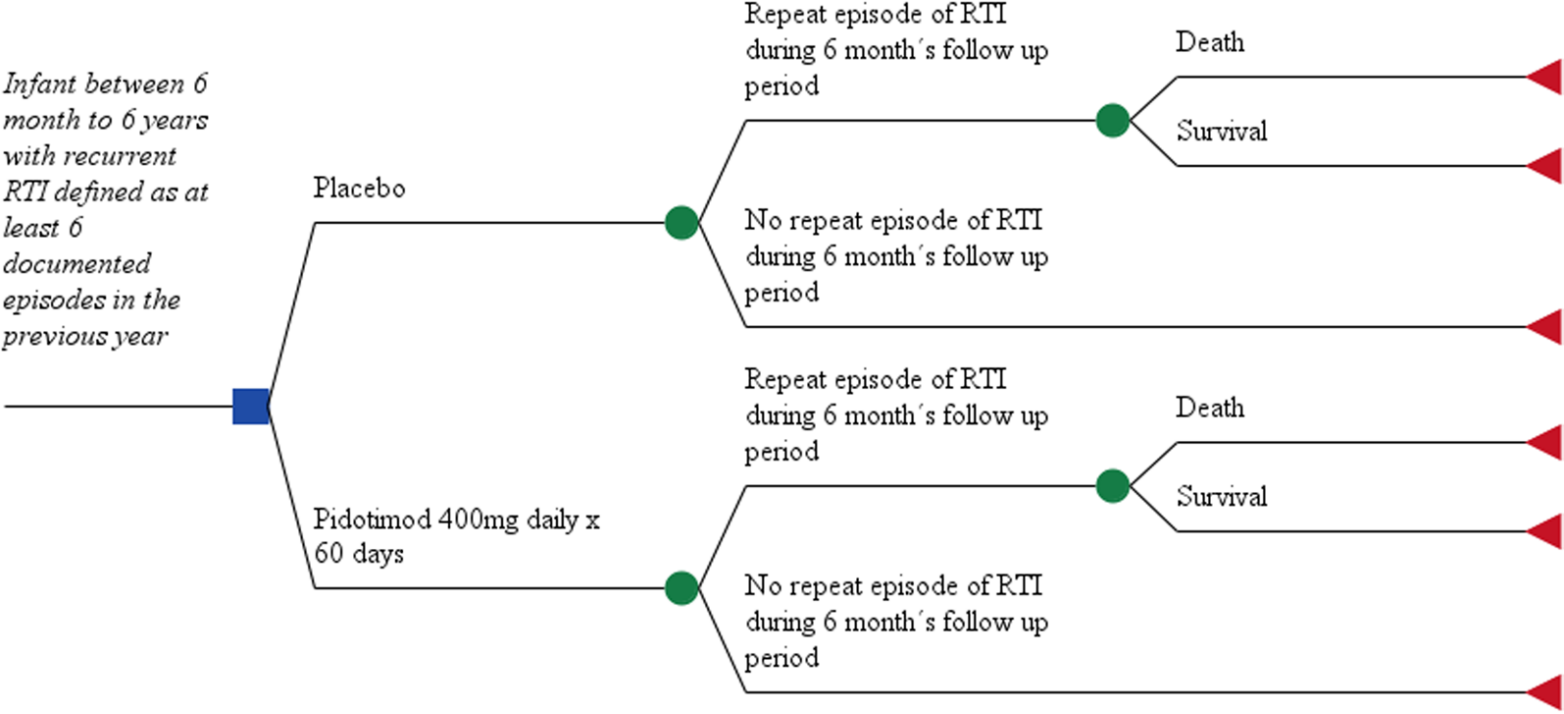
Decision tree model

A decision tree model was used to estimate the cost and quality-adjusted life-years (QALYs) of Pidotimod as a preventive treatment of RTI. It was decided to use a decision tree model because we are going to model interventions (Pidotimod vs placebo) with an outcome (the incidence of new episodes of RTI at six months after of the onset of this drug) that can be measured at a specific time point. This decision tree model was constructed according to the natural history of RTI, Fig. 1. The base case correspond a patient aged 1–6 years with a history of recurrent RTIs; defined as at least 6 documented episodes in the previous year. The decision tree begins with a decision node in which there are two options: Placebo or PDT 400 mg daily for 60 days. Then, in both decision nodes, there are two possibilities that the patient dies or survives this new episode of RTI. The only difference between the two subtrees is the probability of new episode of RTI at 6 months. This probability in the on the subtrees with PDT is lower than in the branch placebo because it was multiplied by the relative risk of this intervention as detailed later.

The time horizon defined was six months. Given the short time horizon, no discount rates were applied to costs or QALYs. Cost-effectiveness was evaluated at a willingness-to-pay (WTP) value of US$5180 [6]. In Colombia there is no data on the frequency of recurrent pneumonia in the study population. For this purpose, the proportion of recurrent pneumonia reported in the control group of the meta-analysis by Niu et al. was taken as 33% to 37% according to the follow-up period [5]. The authors agreed a Threshold of 20%, taking into account the incidence of severe lower respiratory infection reported in Colombia in the bibliographic citations, which according to the age group range from 42% in children under 1 year of age to 8% in children between 6 and 10 years of age [7–9]. Since Niu's meta-analysis does not report mortality, the mortality due to pneumonia in children under 5 years of age reported in the national vital statistics system was taken, and a sensitivity analysis was performed to estimate the impact on the incremental cost-effectiveness ratio in the case of values higher than the one used [7–9].

The relative risk and probability of RTI at 6 months were extracted from recent systematic review and meta-analysis aimed to assess the effectiveness and safety of pidotimod (PDT), an immunostimulant, in treatment of RTI in children The authors made a search of RTC in PubMed, EMBASE, Web of Science, Cochrane Library, ClinicalTrials.gov, CBM and CNKI from their inception up to February 2018. All randomized controlled trials using PDT with various treatment durations and enrolling pediatric patients were included. The interventions were PDT plus conventional treatment (e.g., anti-bacterial and antiviral therapy) or PDT alone versus the conventional treatment plus placebo or conventional treatment alone. A total of 29 RCTs consisting of 4344 pediatric patients were included in this meta-analysis. Ten RCTs were published from Italy, Russia or Greece, and 19 RCTs were published by Chinese groups. PDT was associated with a significant increase in the proportion of participants who had lower RTIs (RR 1.59; 95% CI 1.45–1.74, *p* < 0.00001); without increase the risk of adverse events of any cause (RR = 1.05, 95% CI 0.72–1.54, *p* = 0.80) [5].

Utilities were extracted from a utility assessment study of parent preferences for pediatric health outcomes [10], Table 1. In this study was conducted in 4016 parents or guardians at least 18 years of age with at least 1 child under age 18 years, each subject's utilities were assessed on 3 random health states out of 29 chosen for the study. We assumed that the utility of the new RTI state is equal to the utility reported for the 10-day hospitalization state in this study, given that the worst-case scenario of a new RTI, such as uncomplicated pneumonia, requires hospitalization rather than outpatient management. For the patient who did not develop RTI, it was assumed that the utility of this condition is equal to that reported for uncomplicated acute otitis media; given that the base case are patients with previous RTI and do not have a totally healthy state of health in a period of 6 months. Both the time trade-off and standard gamble methods were used to measure utilities.

**Table 1:** Model inputs

Model input	Base case value	Distribution
*Probabilities*		
Repeat episode of RTI	0.20	β (SD: 0.05)
Mortality Repeat episode of RTI	0,008	β (SD: 0.001)
*Utility*		
Case Base	0.94	β (SD: 0.01)
Repeat episode of RTI	0.87	β (SD: 0.2)
*Cost*		
Repeat episode of RTI (US$)	2022	Γ (SD: 505)
Pidotimod × 60 Day (US$)	196	Γ (SD: 49)
*Pidotimod effectiveness*		
Reduction of probability of Repeat RTI	0,6	LogN (SD: 0.03)

*RTI* Respiratory tract infections

Since utilities and relative risks do not come from the Colombian population, they were subjected to probabilistic sensitivity analysis as detailed below as recommended by Consolidated Health Economic Evaluation Reporting Standards (CHEERS) Statement [11]. We did this analysis from a societal perspective (including direct and indirect costs). All direct and indirect costs were extracted from a previously published cost-illness study in children with pneumonia in Colombia [12].

This study, using a micro costing methods, estimated the direct medical costs and indirect non-medical costs of pneumonia in children under 5 years, based on information obtained from 275 patients hospitalized using the databases of the Individual Registry of Services Provision and Sufficiency identifying pneumonias and bronchiolitis that lead to hospitalization and/or death, as well as the costs experienced by caregivers of these patients, defined as well as the costs experienced by the caregivers of these patients, defined as direct non-medical direct non-medical costs, out-of-pocket expenses and indirect costs, obtained through a survey a survey designed, validated and implemented for this study [12]. In this study, using the bootstrapping technique, 50,000 iterations of direct medical costs, direct non-medical costs and indirect costs were generated for the estimation of the respective confidence intervals for each of these data. Drug costs were taken from the National Drug Price Information System (SISMED, 2021) [13]. All cost costs were transformed to 2021 costs using official inflation data in Colombia. We used US dollars (Currency rate: US$ 1.00 = COP$ 3,900) to express all costs in the study [8]. The incremental cost-effectiveness ratio (ICER) was calculated using the following formulae:

$$ICER = \frac{{\begin{array}{*{20}c} {Expected\,annual\,cost\,per\,patient\,with\,PDT\,-\,} \\ {Expected\,annual\,cost\,per\,patient\,without\,PDT } \\ \end{array} }}{{\begin{array}{*{20}c} {QALY\,per\,patient\,with\,PDT\,-\,} \\ {QALY\,per\,patient\,without\,PDT } \\ \end{array} }}$$

Also, we estimated the net monetary benefit (NMB). NMB represents the value of an intervention in monetary terms [14]. NMB is calculated as (incremental benefit x threshold)  − incremental cost. Incremental NMB measures the difference in NMB between alternative interventions, a positive incremental NMB indicating that the intervention is cost-effective compared with the alternative at the given willingness-to-pay threshold.

### Sensitivity analysis

We conduct a one-way sensitivity presenting these results in the tornado diagram. Probabilistic sensitivity analysis was also performed. For this purpose, random sampling was performed from each of the parameter distributions. We used the beta distribution utilities, the gamma distribution for costs, and log normal for relative risg, see Table 1. For each treatment strategy, we calculated the expected costs and QALYs using the combination of all parameter values in the model. To do this calculation, a second-order Monte Carlo simulation with 10,000 replications of each parameter was made: resulting in the expected cost-utility for each treatment strategy. To represent decision uncertainty, we plot the cost-effectiveness and acceptability frontiers. TreeAge Pro Healthcare 2022 software® was used in all analyses.

## Results

The main results are presented in Table 2. The base-case analysis showed that compared with placebo, PDT was associated with lower costs and higher QALYs. The expected annual cost per patient with PDT was US$797 (CI 95% US$794–US$801) and with placebo was US$1175 (CI 95% US$1169–US$1181). The QALYs per person estimated with PDT was 0.95 (CI 95% 0.94–0.95) and with placebo was 0.94 (CI 95% 0.94–0.94). The NMB with PDT was US$ 4121 (CI 95% 4114–4127) and with placebo was US$ 3710 (CI 95% 3700–3720). This position of absolute dominance (PDT has lower costs and higher QALYs than placebo) of PDT made it unnecessary to estimate the incremental cost-effectiveness ratio.

**Table 2:** Cost effectiveness analysis

Strategy	Cost,US$ (IC95%)	Diff ($)	QUALYs (IC95%)	Diff (QALYs)	NMB(US$ (IC95%)
Podotimod	797 (794–801)		0,95 (0,94–0,95)		4121 (4114–4127)
Placebo	1175(1169–1181)	378	0,94 (0,94.9,94)	0,01	3710 (3700–3720)

### Sensitivity analysis

**Fig. 2:**
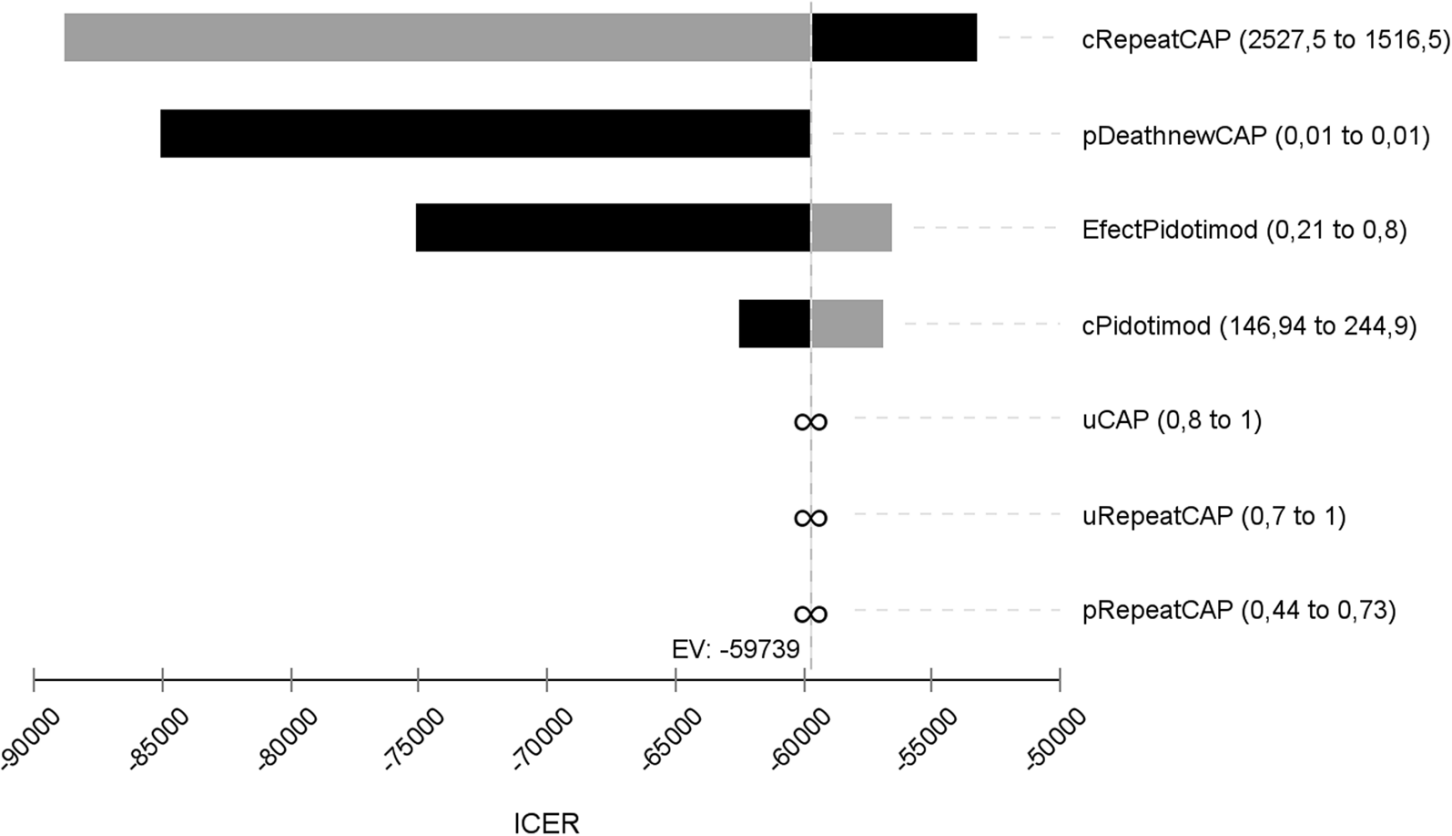
Tornado diagram. CRepeatCAP: Cost of new episode of respiratory tract infection, pDeathnewCap: mortality or new episode of respiratory tract infection, EfectPidotimod: relative risk of pidotimod, cPidotimod: cost of pidotimod, Ucap: utility of respiratory tract infection, uRepeatCAP: utility of new episode of respiratory tract infection, PRepeatCap: probability of new episode of respiratory tract infection

**Fig. 3:**
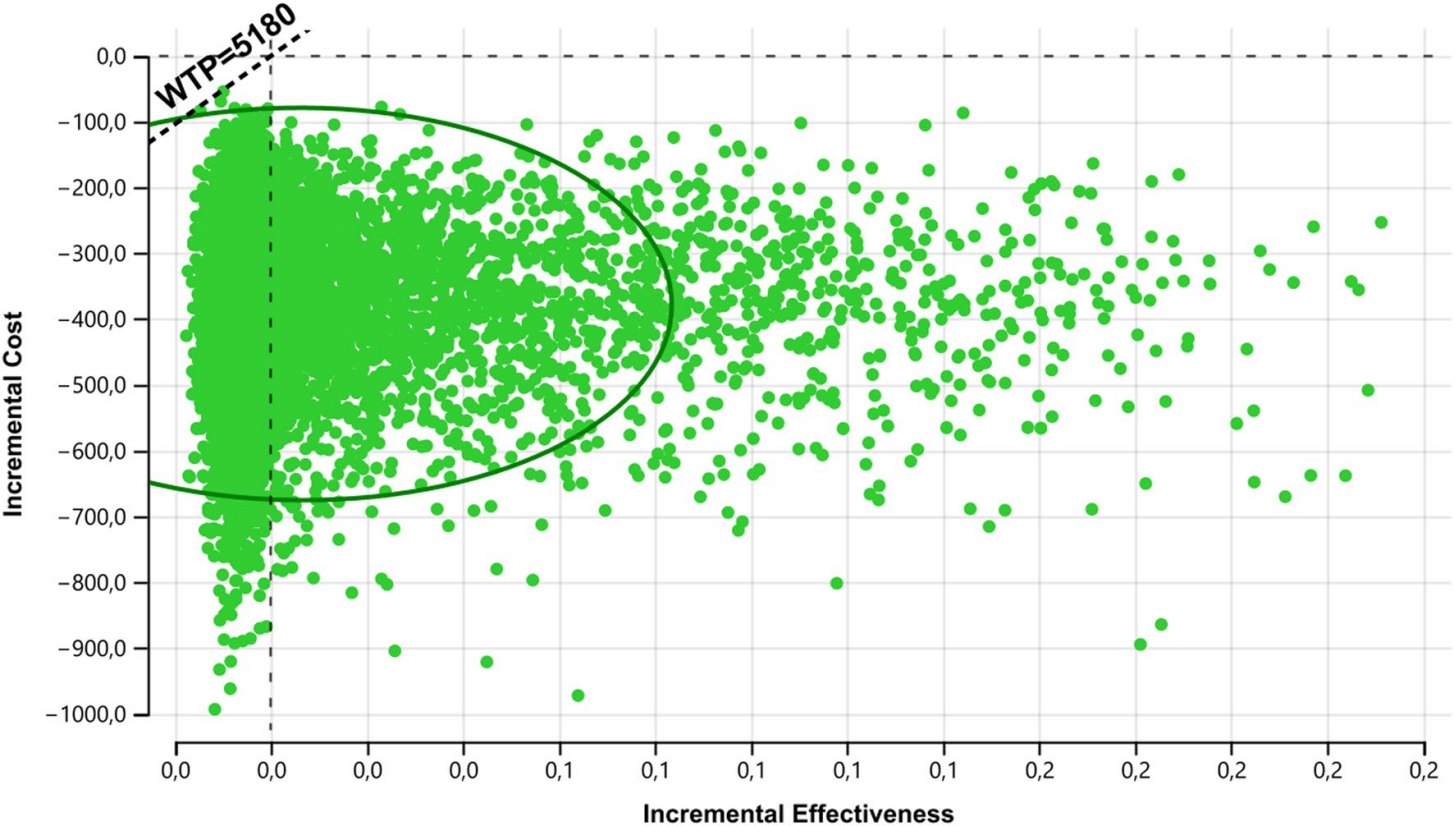
Cost effectiveness plane

**Fig. 4:**
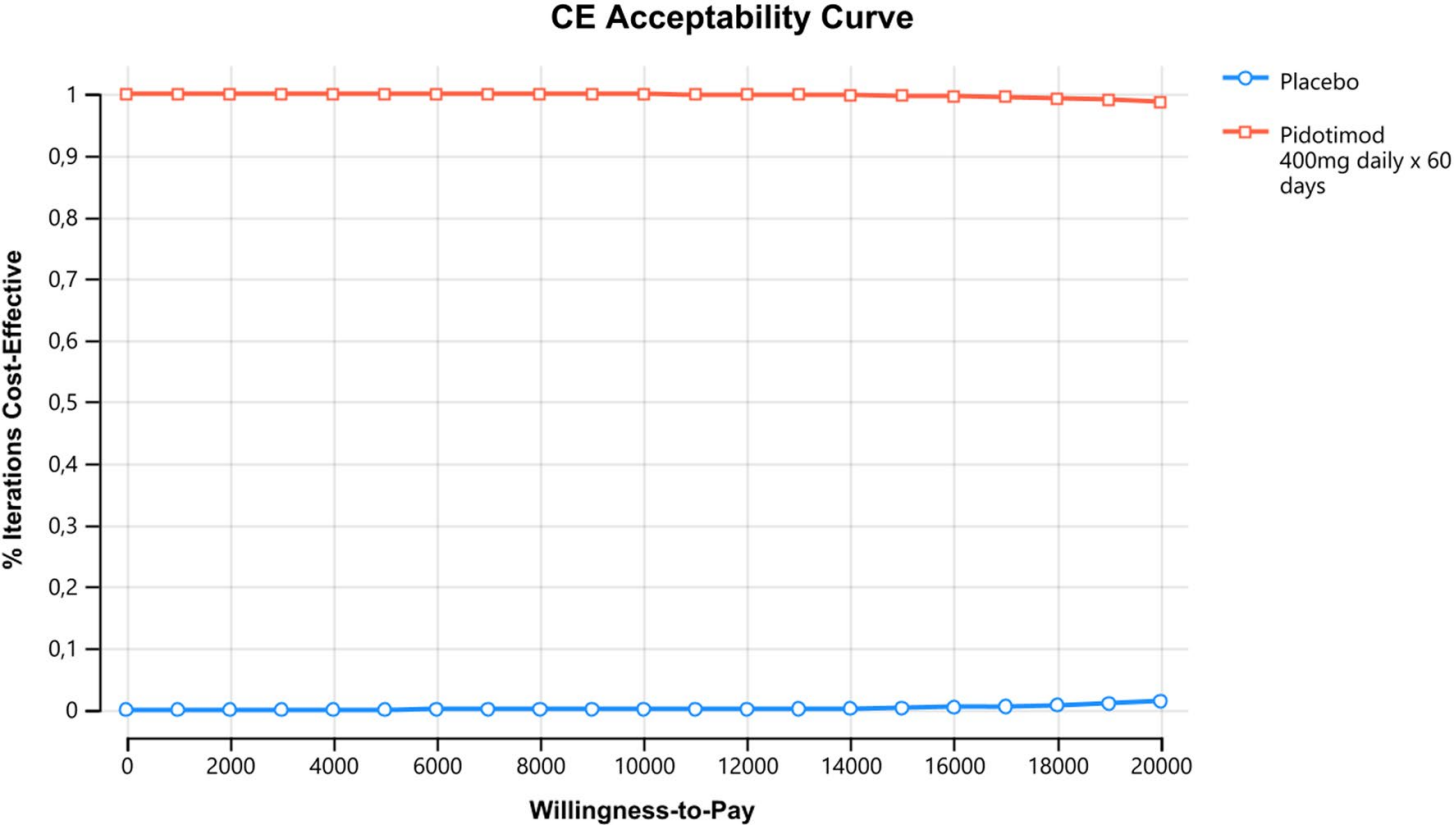
Acceptability curve

In the deterministic sensitivity analyses, our base‐case results were robust to variations in utilities, probabilities, relative risk, and cost; Fig. 2. That is, changing each of the parameters, within the ranges mentioned in the methods section, of cost, profit, transition probabilities and relative risk did not alter the incremental cost-effectiveness ratio significantly or change its interpretation. The results of the probabilistic sensitivity analysis are graphically represented in the cost-effectiveness plane, Fig. 3. This scatter plot shows that 99% of simulations the ICER were below WTP in quadrants 2 (42%) or 3 (57%). The incremental net monetary benefit (INMB) calculated in the second-order Monte Carlo simulation was US$410 (CI 95% US$406–US$414). This positive value of INMB means that the incremental benefits in monetary terms for the WTP are higher than incremental costs of this drug in Colombia; thus, this medication can be declared as cost-effective. For WTP in Colombia (US$5180 per QALY), the PDT is cost-effective in 100% of cases versus placebo. as can be seen in the acceptability curve Fig.4.

## Discussion

Our economic evaluation shows that PDT is cost-effective to reduce the incidence rate of recurrent respiratory tract infections in children in Colombia. Evaluating treatments to reduce costs and optimize health resources is a priority for all health systems, especially in RTI, which, due to their frequency, generate a high economic burden in developing countries. In our study, PDT was a strategy that generated savings US$410 (CI 95% US$406 to US$414) per patient, which is not insignificant given the frequency of RTI in most developing and developed countries.

To the best of our knowledge, there are no previously published economic evaluations of PDTs. The closest comparator has been another immunostimulant substance, OM-86 BV. Our results are in line with previous economic evaluations of OM-85. Xuan et al., using cost-effectiveness decision tree model compare OM-85 with placebo therapy for managing the acute exacerbation of chronic bronchitis and rhinosinusitis in the Chinese population From a Chinese payer perspective [15]. OM-85 is a cost-effective therapy in China. OM-85, when compared with placebo arm, can prevent one additional episode exacerbation of rhinosinusitis with only RMB 1182.84 extra costs, below of WTP in China. Troriano et al., in a systematic review and metanalysis of 13 studies found that OM-85 BV is associated with a reduction in the mean number of COPD exacerbations (*p* < 0.01; WMD = − 0.86; CI 95%: − 1.38, − 0.34) and in the days of antibiotic therapy (*p* < 0.01; WMD = − 9.49; CI 95%: − 11.93, − 7.05) [16]. Perssey et al., using a decision-analysis model, evaluated the pharmacoeconomic impact for the French Social Security System of preventing recurrent acute rhinopharyngitis in at-risk children with OM-85 BV [17]. Using OM-85 BV prevention, 1.52 infections were prevented in 6 months saving 67.83 Euro on the costs of care for the recurrently infected child. OM-85 was cost-effective in preventing ARI and also showed cost savings in over 70% of cases for direct costs with a reduction of 2.61 episodes of ARI during a follow-up of six months [18]. These results with OM-85 have been similar to those obtained in other countries such as France and Italy [17, 19, 20].

There is no consensus concerning interventions to prevent RTI. Reducing exposure to damp and mold, for example, is the intervention for which a good-quality systematic review supports the elimination of this risk factor for recurrent respiratory acute infections [4]. Other environmental interventions with low or very low-quality studies are also recommended, such as discouraging exposure to second and third-hand smoke and pollutants in general, in addition, to improving handwashing as one of the best methods to reduce respiratory infections [4, 21]. Our study provides further evidence regarding the efficiency of use of this PDT in children with RTI, which complements the growing evidence of effectiveness and safety of this drug.

Our study has some limitations. Some inputs they do not come from Colombian population, such as risk, utilities, transition probabilities and were extracted from the literature. However, all these data were subjected to deterministic and probabilistic sensitivity analyses. It was confirmed that none of them in the evaluated ranges change the final conclusion of the study.. Since the costs were estimated retrospectively, information bias cannot be ruled out. However, they were also evaluated in the sensitivity analysis, and the estimated ranges do not change the final conclusion of the study.

## Conclusion

In conclusion, our study shows that PDT is a cost-effective strategy to reduce the incidence rate of recurrent respiratory tract infections in children. Our study provides evidence that should be used by decision-makers to improve clinical practice guidelines.

## Data Availability

Zenodo. https://doi.org/10.5281/zenodo.5895163

## Abbreviations

RR: Relative risk
RTC: Randomized controlled trial
OR: Odds ratio
CAP: Community‐acquired pneumonia
WTP: Willingness-to-pay
QALY: Quality adjusted life years
ICER: Incremental cost effectiveness ratio
